# Catatonia: Clinical Overview of the Diagnosis, Treatment, and Clinical Challenges

**DOI:** 10.3390/neurolint13040057

**Published:** 2021-11-08

**Authors:** Amber N. Edinoff, Sarah E. Kaufman, Janice W. Hollier, Celina G. Virgen, Christian A. Karam, Garett W. Malone, Elyse M. Cornett, Adam M. Kaye, Alan D. Kaye

**Affiliations:** 1 Department of Psychiatry and Behavioral Medicine, Louisiana State University Health Shreveport, 1501 Kings Hwy, Shreveport, LA 71103, USA; sarah.kaufman@lsuhs.edu (S.E.K.); janice.hollier@lsuhs.edu (J.W.H.); 2 Department of Anesthesiology, College of Medicine-Phoenix, University of Arizona, Phoenix, AZ 84006, USA; celinav@email.arizona.edu; 3 School of Medicine, Louisiana State University Health Shreveport, Shreveport, LA 71103, USA; cjk002@lsuhs.edu (C.A.K.); gwm002@lsuhs.edu (G.W.M.); 4 Department of Anesthesiology, Louisiana State University Shreveport, Shreveport, LA 71103, USA; elyse.bradley@lsuhs.edu (E.M.C.); alan.kaye@lsuhs.edu (A.D.K.); 5 Department of Pharmacy Practice, Thomas J. Long School of Pharmacy, University of the Pacific, Stockton, CA 95211, USA; akaye@pacific.edu

**Keywords:** schizophrenia, catatonia, benzodiazepines, ECT

## Abstract

Catatonia is a syndrome that has been associated with several mental illness disorders but that has also presented as a result of other medical conditions. Schizophrenia and other psychiatric disorders such as mania and depression are known to be associated with catatonia; however, several case reports have been published of certain medical conditions inducing catatonia, including hyponatremia, cerebral venous sinus thrombosis, and liver transplantation. Neuroleptic Malignant Syndrome and anti-NMDA receptor encephalitis are also prominent causes of catatonia. Patients taking benzodiazepines or clozapine are also at risk of developing catatonia following the withdrawal of these medications—it is speculated that the prolonged use of these medications increases gamma-aminobutyric acid (GABA) activity and that discontinuation may increase excitatory neurotransmission, leading to catatonia. The treatment of catatonia often involves the use of benzodiazepines, such as lorazepam, that can be used in combination therapy with antipsychotics. Definitive treatment may be found with electroconvulsive therapy (ECT). Aberrant neuronal activity in different motor pathways, defective neurotransmitter regulation, and impaired oligodendrocyte function have all been proposed as the pathophysiology behind catatonia. There are many clinical challenges that come with catatonia and, as early treatment is associated with better outcomes, it becomes imperative to understand these challenges. The purpose of this manuscript is to provide an overview of these challenges and to look at clinical studies regarding the pathophysiology, diagnosis, and treatment of as well as the complications and risk factors associated with catatonia.

## 1. Introduction

Catatonia is a syndrome that has been associated with several mental illness disorders but that has also presented with other medical conditions. It is defined as a group of symptoms that involve a lack of movement as well as a lack of communication. It can be accompanied by agitation, confusion, and restlessness. It is a complex psychomotor syndrome that was previously seen as a subtype of schizophrenia; however, with changes to the DSM-5, it can now be classified as stemming from other mental health disorders, including brief psychotic, schizophreniform, and schizoaffective disorders, as well as having been induced by a substance [1]. It is most commonly associated with bipolar disorder. It may also be diagnosed independently of any syndromes or medical illness [2].

Catatonia was first categorized by Karl Kahlbaum as its own entity in 1874 [3]. Emil Kraepelin’s description of catatonia differed from Kahlbaum’s in that he attempted to tie catatonia into his vision of dementia praecox [3]. This is the collection of symptoms that characterized early definitions of schizophrenia. Eugen Bleuler brought Kraepelin’s idea that catatonia equated schizophrenia to the United States and even indicated this his textbook, which was written in 1916 [3]. Bleuler took it a bit further and thought that a patient with catatonia was suppressing unpleasant memories by “silence, tenseness and rigidity, refusal to obey commands, and displacing rising emotion and tensions into motor acts that shut out reality” [3]. Although it was often previously categorized with schizophrenia or associated with other mental health disorders and neurological disorders, new changes in the DSM-5 have opened up an opportunity for it to be recognized independently of these conditions. This is a huge step forward, as catatonia is not just associated with psychiatric disorders.

Catatonia has a complex presentation that is composed of multiple signs and symptoms, of which only three need to be present for diagnosis. It may be thought of as occurring with schizophrenia or with mania; however, patients in other settings with various general medical health conditions may develop an episode of catatonia. It is essential to identify catatonia early on for treatment to protect the patient from developing any further complications. A number of medical conditions can mask catatonia, delaying its treatment.

Some general medical conditions may also present with catatonic symptoms or place patients at a higher risk of catatonia, and several case reports have been published on the subject. These case reports include conditions such as hyponatremia, cerebral venous sinus thrombosis, and liver transplantation, among others [4,5,6]. Patients taking benzodiazepines or clozapine are also at risk of developing catatonia after withdrawal from these medications [7]. It is speculated that the prolonged use of these medications increases GABA activity and that discontinuation may increase excitatory neurotransmission, leading to catatonia [7]. Despite this reaction, benzodiazepines are still used as the main form of treatment for catatonia.

The diagnostic criteria for catatonia in the current DSM-5 require three or more of the following symptoms: stupor, waxy flexibility, catalepsy, mutism, posturing, negativism, stereotypes, mannerisms, grimacing, agitation, echopraxia, and echolalia [8]. These diagnostic criteria apply to both adults and children, but in children, catatonia often presents as a result of somatic conditions or substance use. Catatonia associated with schizophrenia in children is considered serious and needs immediate attention, as there is a greater opportunity for poor prognosis [8]. Patients who develop catatonia accompanied with acute autonomic instability have an increased risk of complications and mortality [9,10]. This autonomic instability can be suggestive of a cause that needs immediate treatment. The treatment of catatonia often involves the use of benzodiazepines, such as lorazepam, that can be used in combination therapy with antipsychotics to minimize symptoms [11]. Definitive treatment may be found with electroconvulsive therapy (ECT), which provides patients with short electrical brain stimulation under anesthesia. ECT has been proven to be effective in the treatment of catatonia, especially with early initiation [11].

Given the various presentations and symptoms for catatonia, it can be challenging to diagnose and to treat it if not identified correctly and in a timely manner. Catatonia may be underdiagnosed in populations with mental illness or general medical conditions. Although the changes made in the DSM-5 aim to address the under-recognition of this condition and the discrepancies previously noted in the DSM-4, its presentation may still be missed. It is essential for healthcare professionals to consider it as part of the differential diagnosis in those presenting with criteria that meet the diagnosis of catatonia. This manuscript will look at the different causes and the management of catatonia.

## 2. Catatonia Causes, Presentation, and Pathophysiology

### 2.1. Types of Catatonia

There are three types of catatonia that clinicians need to be aware of. The first, and most common, is akinetic catatonia. A patient with this type of catatonia will stare and appears to be non-responsive [12]. Response to vocal and noxious stimuli is decreased [9]. These patients are alert and aware of their surroundings. The second type of catatonia is excited catatonia. A patient with this type may move, but their movements seem pointless and impulsive. They can appear agitated, combative, or even delirious [12]. The excess motor activity could cause either harm to the patient themself or harm to others [9]. The last type is malignant catatonia. This type of catatonia is dangerous and is associated with autonomic instability [12]. This can be seen in neuroleptic malignant syndrome and can signal a potential lethal underlying cause of the catatonia. Malignant catatonia can evolve rapidly, within a matter of days [13]. It is because of this rapid evolution that clinicians need to keep this in mind when seeing a person with suspected catatonia and act quickly to treat the underlying cause.

Although not an official subtype of catatonia, periodic catatonia can present as a diagnostic challenge for clinicians and should be discussed. Periodic catatonia is a rare form of catatonia where the symptoms present in phases and can disappear completely in between episodes [14]. The pathophysiology of periodic catatonia is unclear at this time, though it may be related to a dysfunctional GABA signal since acute cases respond well to benzodiazepines [15]. There have been cases reported where treatment with an atypical antipsychotic relieved symptoms [15].

### 2.2. Causes

It is important to note that catatonia is a constellation of symptoms that are a result of an underlying disorder. Catatonia itself is not a disorder but rather a syndrome. Psychiatric disorders are the first source that comes to mind when thinking about the underlaying causes of catatonia. Patients with bipolar disorder, autism, schizophrenia, major depressive disorder, or mixed psychiatric conditions all have a higher incidence of catatonia than the general population [16]. In fact, roughly 35% of individuals with schizophrenia will show symptoms of catatonia at some point [17]. It is because of this that it is important to keep catatonia in mind in patients with schizophrenia when abnormal movement and communication is present, as this could be a presentation of neuroleptic malignant syndrome, a life-threatening syndrome that can be caused by antipsychotic use. Roughly 20% of patients with catatonia have a medical cause rather than a psychiatric one [18]. Additionally, general medical conditions such as strokes, neoplasms, infections, autoimmune disorders, neurodegenerative diseases, metabolic derangements, and certain drugs have all been associated with catatonia [18]. Infectious and autoimmune etiologies account for roughly 29% of cases associated with general medical causes, and studies have shown that meningitis and encephalitis as well as systemic bacterial, viral, or fungal infections may result in catatonia [19]. Further, autoimmune processes, particularly N-methyl-D-aspartate receptor (NMDAR) encephalitis and systemic lupus erythematous (SLE) also have a strong association with catatonia [19]. In fact, NMDAR encephalitis is responsible for 72% of all autoimmune cases of catatonia. The percentages presented in this section are meant to highlight that catatonia should be on the clinician’s radar whenever the syndrome is even slightly suspected. The exact reason why some medical conditions lead to catatonia is not well understood; however, direct neurotoxic effects, the patient’s psychological reaction to the insult, or mediation by acute phase reactants have all been suggested as potential causes [19,20].

Another interesting theory of the cause of catatonia seen in the literature is catatonia that happens in the face of extreme fear. There is a theory that catatonia is originally derived from encounters with predators whose attack instincts were based on movements [21]. The thought is that catatonia could be an end state response to the feelings of imminent doom [21]. A study in an elderly population found that catatonic patients experienced more anxiety and hyperactivity [22]. This also may lend credibility to the notion that anxiety and fear can be part of the causes of catatonia, although catatonia is more complex and has more probable causes.

### 2.3. Pathophysiology

The pathophysiology of catatonia is not currently well understood. However, recent studies suggest that three motor pathways within the brain and brainstem are responsible [23,24,25]. The first pathway leads from the primary motor cortex (M1) to the putamen, the internal and external pallidum, the thalamus, and then back to M1. This pathway is responsible for the inhibition and excitation of movements [24,25]. Another circuit runs between the M1, thalamus, cerebellum, and pontine nuclei and is responsible for motor dynamics and timing [23,24,25]. Lastly, the third circuit is composed of the M1, supplementary motor area (SMA), posterior parietal cortex, and medial prefrontal cortex and controls motor organization and speed [23,24,25]. The dysfunction of any of these circuits could lead to catatonic symptoms. Blood flow to the M1 and SMA has been shown to be increased in patients with catatonia compared to those without catatonia, further suggesting the increased neural activity of these circuits, likely resulting in catatonic behavior [26,27,28,29].

Reduced GABA activity, specifically GABA-A receptor activity, in the right lateral orbitofrontal and right posterior parietal cortex is thought to be another driver of the dysfunction seen in catatonia syndrome [30]. This dysfunction could explain the motor and affective symptoms seen in catatonia. This would explain why patients with catatonia respond well to and why benzodiazepines remain the mainstay of treatment for catatonia. This class of drugs stimulates GABA-A binding and relieves the symptoms of catatonia, presumably by lowering the increased neural activity in the circuits described above. Dysfunctional connections between the orbitofrontal cortex and the medial prefrontal cortices can be partially reversed by the administration of benzodiazepines, and imaging shows reduced GABA-A receptor density in cortical areas such as the left sensorimotor cortex [31,32]. Therefore, dysfunctional GABA-A signaling also seems to contribute to catatonia.

Not only are GABA-A receptors associated with catatonia, but the excitatory glutamatergic N-methyl-D-aspartate receptor (NMDAR) appear to be associated as well. Glutamate abnormalities have been seen in the basal ganglia [33]. Glutamate hyperactivity is thought to be the cause of catatonia symptoms in this case. As previously stated, NMDAR encephalitis is strongly associated with catatonia, even more so than it is with psychosis [19]. During this inflammatory process, NMDARs are internalized into cells, and overall levels of this receptor are decreased [34,35]. Therefore, the dysfunction of both the NMDAR and GABA-A receptors has been implicated in the pathogenesis of catatonia [33].

Dopamine dysfunction has also long been postulated as a cause for catatonia symptoms. There is some evidence that the potency of a dopamine D2 receptor blockage is directly related to the risk of exacerbating catatonia or even provoking malignant features [36]. This can be seen in neuroleptic malignant syndrome, which can manifest as catatonia. A strong dopamine blockade is thought to cause the symptoms seen in this syndrome. However, another thought is that there is a balance of GABA-A and dopamine that must be maintained in the mesostriatal and mesocortiolimbic systems as well as in the hypothalamus. When this system is dysfunctional, the vulnerability to catatonia emerges from the use of dopamine antagonists [37]. This particular theory of the pathophysiology of catatonia as well as GABA and glutamate dysfunction does not fully explain the presentation seen in patients.

Other autoimmune disorders can present with catatonia. T-cell mediated disorder, such as acute demyelinating encephalomyelitis, can also present with catatonia [19]. Catatonia is also associated with autoimmune encephalopathies that are associated with antineuronal antibodies. These antibodies become internalized and cause the neuron to cease to function [19]. These disorders suggest that the immune system can play an important role in the pathogenesis of catatonia and need to be considered when a differential diagnosis is formulated.

Studies have shown that catatonia is substantially heritable and that patients have a roughly 27% risk of experiencing catatonic symptoms if a first-degree relative has been affected [38,39,40]. One gene that is implicated in the heritability of catatonia is *CNP*, which codes for cyclic nucleotide phosphodiesterase, an enzyme important for oligodendrocyte function and myelination [41]. In mouse models, knockout of this gene leads to a catatonic and depressive phenotype in the affected mice [42]. Studies have found that loss-of-function mutations in this gene are more prevalent in schizophrenic patients with catatonia compared to schizophrenics without catatonia [43]. Taking all of these pieces together, we can infer that aberrant neuronal activity in different motor pathways, defective neurotransmitter regulation, and impaired oligodendrocyte function may all be part of the pathophysiology of catatonia.

### 2.4. Presentation

The presentation of catatonia is varied. In general, catatonia can be behavior that is either increased, decreased, or abnormal compared to baseline, and the DSM-5 does not provide a separate diagnostic code for catatonia as a distinct psychiatric condition [44]. As per the DSM-5, to diagnose catatonia, three of the following twelve symptoms must be present: stupor (no psychomotor activity; not actively relating to the environment), catalepsy (passive induction of a posture held against gravity), waxy flexibility (slight, even resistance to positioning by the examiner), mutism (no, or very little, verbal response (excluded if known aphasia)), negativism (opposition or no response to instructions or external stimuli), posturing (spontaneous and active maintenance of a posture against gravity), mannerism (odd, circumstantial caricature of normal actions), stereotypy (repetitive, abnormally frequent, non-goal-directed movements), agitation, grimacing, echolalia (mimicking another’s speech), and echopraxia (mimicking another’s movements) [44,45,46].

The Bush Francis Catatonia Rating Scale (BFCRS) overlaps with the DSM-5 criteria and also adds other presentations, including ambitendency (appearance of being stuck in indecisive or hesitant movement), automatic obedience (mechanical and reproducible compliance with examiner’s request, even if dangerous), autonomic abnormality (diaphoresis, palpitations, or abnormal temperature, blood pressure, pulse, or respiratory rate), combativeness (striking out against others with or without potential for injury), gegenhalten (resistance to positioning by examiner that increases proportionally to applied force), grasp reflex (strong grasp of any object in proximity of the hand or upon touch), impulsivity (patient suddenly engages in inappropriate behavior without provocation; afterwards, can give no or only a facile explanation), mitgehen (exaggerated movements in response to light pressure), perseveration (whole or partial repetition of actions or verbal content that is not goal directed), rigidity (resistance by way of increased muscle tone), staring, verbigeration (continuous, directionless repetition of words, phrases, or sentences), and withdrawal (no eye contact, refusal to take food or drink when offered, or both; turning away from examiner or social isolation) [47]. The BFCRS has a total of 23 items that are tallied to give an overall score. The presentation of a patient with catatonia is not fixed and may vary from interview to interview, and patients are often cachectic and disheveled [48].

Autonomic instability can also be seen in catatonia. This type of catatonia has been labeled “malignant catatonia.” This can be due to an underlying condition that can be life-threatening. Liable blood pressures have been seen in benzodiazepine withdrawal, and case reports have illustrated that catatonia has also been associated with this withdrawal [49]. These symptoms are thought to be due to the disruption of the GABA system as well as dopamine disturbances. GABA has been associated with the increased firing of dopamine cells in the ventral tegmental area and increased metabolism in the striatum [49]. The thought is that the withdrawal of GABA can lower the dopamine and acetylcholine ratio, which could cause catatonia [49]. This can lead to the death of the patient, so a high index of suspension should be kept if autonomic instability is present in the presence of catatonia for possible withdrawal.

## 3. Catatonia Current Treatment

The early initiation of treatment in patients presenting with catatonia can reduce the risk of complications. When patients develop catatonia, their risk of developing deep venous thrombosis and pulmonary embolism increases substantially and occurs frequently [50]. This is due to the patient’s immobility. Other complications include malnutrition, infection, and muscle contractures, as the patient’s mobility is decreased, and they may refuse oral intake [51,52,53]. Despite the development of possible complications, most patients experience a resolution of symptoms with proper management [54]. As stated in previous sections, autonomic instability may unmask an underlying condition that is also causing the catatonia. The treatment of that condition is critical, as autonomic instability may be part of a life-threatening condition.

The first-line treatment for catatonia is generally benzodiazepines, unless malignant catatonia is present. Benzodiazepines work on the GABA-A receptors and help to relieve the GABA dysfunction seen in some patients presenting with catatonia syndrome. Various benzodiazepines have been studied, and while lorazepam is typically used, others can also be considered, especially when additional symptoms or disorders are present. Caution must be used in patients exhibiting delirium, as their presentation can worsen; such patients may require lower doses for treatment [55]. When catatonia is suspected, a lorazepam challenge can be performed. This is done by giving a dose of lorazepam, either through IM or IV, and watching for a response. A response indicates the need for high suspicion of catatonia. In the case of substance-induced catatonia, a combination of lorazepam and diazepam has been shown to be an effective treatment plan, with the resolution of symptoms occurring within a day [56].

The starting dose of lorazepam is 2 to 6 mg/d and can be increased up to 12 to 16 mg/d [54]. With an appropriate dose, a response is usually seen within 3–7 days; however, a treatment response can be gradual and slow in some cases [57]. There is no agreement on how long benzodiazepines should be continued in the treatment of catatonia. However, they are generally discontinued once the underlying illness that was thought to cause the catatonic symptoms remits [57]. However, tapering can cause the catatonia symptoms to return, which necessitates the continuation of benzodiazepines for an unknown amount of time.

ECT has been used to treat various mental disorders for many years and is an established treatment modality that has been proven to be highly effective for several conditions. Treatment involves brief electrical brain stimulations under anesthesia. ECT is a first-line treatment in neuroleptic malignant syndrome, malignant catatonia, and delirious catatonia. ECT is thought to work by increasing cerebral blood flow to the orbitofrontal and parietal cortices, which increases GABA activity and GABA receptor expression [38]. It can also be a definitive treatment when treatment with benzodiazepines has failed. The response rate of catatonia symptoms when ECT is used is around 80–100% [58] It can take several ECT treatments to achieve the desired results [59]. It can take at least six sessions for symptom relief to be seen [38]. The number of total ECT sessions needed cannot be predicted. The termination of ECT can be considered when a full clinical response is achieved or when there is further clinical improvement after two consecutive sessions [58].

Predictors of a favorable response to ECT are noted to be young age, the presence of autonomic dysregulation at baseline, daily ECT during the first week of treatment, longer duration of motor and EEG seizure activity at the final ECT sessions, and less morbidity in the year after ECT [58]. Contraindications to ECT include myocardial infarction within 3 months, elevated intracranial pressure, pheochromocytoma, cerebral tumors, and cerebral aneurysms [60]. Associated side effects may include impaired new learning, anterograde and retrograde amnesia, and autobiographical memory; these side effects usually resolve within weeks to months, but it may take up to 6 months for cognitive function to return to baseline [61,62,63]. A previous history of cognitive impairment places patients at higher risk of developing side effects [61]. If patients experience recurrent episodes, ECT may be continued for maintenance treatment [64].

An emerging form of treatment is Repetitive Transcranial Magnetic Stimulation (rTMS). Similar to ECT, rTMS provides stimulation to the brain, but it does not require patients to undergo anesthesia. rTMS does not produce cognitive side effects and can be used in refractory catatonia for acute or maintenance therapy [65]. This treatment modality can be considered as an option for patients with catatonic schizophrenia but may be restrictive for some patients, as it requires daily appointments for several weeks [65]. Addressing the patient’s co-morbidities and other medical conditions along with catatonia is important in order to provide optimal treatment.

A second-line treatment if benzodiazepine fails and if ECT is either ineffective or refused are medications that modulate glutamatergic activity. These are memantine or amantadine and can be given in twice-daily doses [38]. They are generally well tolerated and can be used either alone or with a benzodiazepine [38]. A final option is the use of an atypical antipsychotic. These should be used with caution, as they can actually worsen catatonia or cause a conversion to malignant catatonia. Atypical antipsychotics should be used in combination with a benzodiazepine, and a low potency agent should be used [38]. In cases such as these, aripiprazole should be considered due to its partial agonist activity [38]. Clozapine is another option that is available for patients with schizophrenia with catatonia [66].

## 4. Clinical Challenges

While ECT has been proven to produce a positive response in patients with catatonia, there are challenging aspects aside from the possible side effects resulting from this treatment modality. One such difficulty is obtaining consent from the patient. Patients in a catatonic state may be unable to provide full consent or refuse such treatment. In the USA, the guardian in these cases may make medical decisions; however, ECT treatment is typically not included, and a petition must be obtained [67]. A catatonic patient’s autonomy sets ethical challenges, as they are unable to fully comprehend the details of ECT treatment [67,68]. This same challenge presents in adolescent patients; however, healthcare professionals must consider the risks versus the benefits [68]. ECT has been found to be safe and effective in the pediatric population and is available as a form of treatment for them.

Other challenging aspects of catatonia involve its various forms of presentation, one of them being periodic catatonia. Periodic catatonia is a subtype of catatonia in which patients regularly experience multiple episodes. Although rare, it is a difficult subtype to diagnose and treat, as it may disappear before it can be treated [14]. Tang et al. describe a case of a 73-year-old woman with episodes of appearing ill for 45 years, which was initially thought to be a non-specific psychosis. She was initially placed on antipsychotics but was noted to have a good response to benzodiazepines. It was later discovered that her symptoms and presentation were consistent with periodic catatonia, and she was treated with multiple sessions of ECT. Her catatonia remained in remission for one year [69]. In patients presenting with multiple episodes of psychosis with symptoms meeting the criteria for catatonia, periodic catatonia should be considered in the differential diagnosis. Treating patients with antipsychotics in such cases may exacerbate catatonia and may induce neuroleptic malignant syndrome or malignant catatonia [70,71]. When neuroleptic malignant syndrome or malignant catatonia occurs, benzodiazepines have been proven to be an effective form of treatment for both conditions [71].

## 5. Clinical Studies

Clinical studies were gathered from PubMed for analysis in this paper. The terms catatonia with the filters for clinical studies and randomized controlled trial were applied to the search. The resulting studies are discussed below and are summarized in Table 1.

### 5.1. Pathophysiology

A double-blind, case–control study was performed to observe the effects of lorazepam on cerebral activity [31]. Patients diagnosed with catatonia were exposed to triggers targeted to elicit positive or negative emotions while researchers used functional magnetic resonance imaging (fMRI) to measure areas of elevated activity in the brain [31]. Images were obtained from catatonic patients after their treatment with 1 mg lorazepam and placebo, respectively. Images under the same conditions were also performed in healthy individuals [31]. Regions of interest (ROIs) in the brain associated with Brodmann Areas were configured, and the intensity of signals in each ROI was measured and compared between groups [31]. When exposed to triggers that elicited negative emotions, fMRI scans showed that catatonic patients experienced a greater decrease in neural activity in the orbitofrontal cortex (OFC) relative to the healthy controls when administered lorazepam rather than placebo [31]. These findings indicate that patients with catatonia may have abnormal function of the OFC and that cerebral GABA signaling may be diminished in catatonic patients [31].

In a double-blind, placebo-controlled crossover study, patients who experienced chronic symptoms of catatonia and who were diagnosed with schizophrenia were recruited to help determine the efficacy of amineptine, a tricyclic antidepressant which also acts as a mixed serotonin and dopamine reuptake inhibitor and releasing agent, in treating catatonia in schizophrenic patients [72]. Amineptine has not been officially approved by the FDA for use in the United States. Each patient was treated for six weeks with either 200 mg amineptine or placebo daily in addition to any medications prescribed before the study began. They then received the alternative treatment for six weeks following a wash-out period of 3 weeks [72]. Patients were psychiatrically evaluated using eight clinical evaluation scales at weeks 0, 6, 9, and 15 [72]. In both patients with catatonic schizophrenia and in healthy individuals, there was no significant decrease in score relative to baseline on any of the clinical scales used when amineptine was administered instead of placebo (*p* > 0.35) [72]. Disregarding a statistically significant but clinically insignificant decrease in negative symptoms, amineptine was not found to effectively treat catatonic symptoms in patients with schizophrenia [72]. These results suggest that the etiology of catatonia in patients with schizophrenia and catatonic symptoms may not be due to altered dopaminergic pathways [72].

Another double-blind, placebo-controlled crossover study was performed to evaluate the efficacy of lorazepam in treating chronic catatonic symptoms in clinically stable schizophrenic patients [73]. Patients were treated daily with 6 mg lorazepam or placebo, and they were then administered the alternate treatment for six weeks following a wash-out period of 4 weeks [73]. Each patient was evaluated for psychiatric symptoms using 12 clinical scales, including the BFCRS and the brief psychiatric rating scale (BPRS), at the beginning of each respective treatment as well as at the third and sixth weeks of each treatment [73]. A significant difference in average score was not observed between patients who received lorazepam or placebo on any of the scales that were used (*p* > 0.05) [73]. There was no clinically significant relief of catatonic symptoms observed in patients who received lorazepam relative to those who received placebo. Lorazepam has been found to be an effective treatment in patients suffering from acute catatonia in previous studies but was shown to be ineffective in treating chronic catatonic symptoms in this study [73,74,75,76]. This implies that patients with schizophrenia who suffer from chronic catatonia may have a different pathophysiology associated with their catatonic symptoms than patients with schizophrenia and acute catatonia [73].

### 5.2. Diagnosis

There are data suggesting that catatonia may be underdiagnosed in adolescent and pediatric populations, which is possibly due to a lack of a universal method for diagnosing a catatonic episode in young patients [77,78]. In a case–control study, researchers addressed the lack of a tool to assess catatonia in adolescent and pediatric populations. In this study, researchers developed the pediatric catatonia rating scale (PCRS) based on established features of catatonia identified in the BFCRS and previous literature [79]. Differing from the BFCRS, the PCRS excludes grimacing, agitation, or impulsivity as well as other signs as components of a catatonic syndrome [79]. However, the PCRS includes six additional components that the BFCRS lacks, such as word salad and incontinence [79]. The validity and reliability of the PCRS were analyzed using 138 psychiatric patients [79]. The PCRS was found to have an Area Under the Curve (AUC) for the Receiver Operating Characteristic (ROC) of 0.983 with a sensitivity greater than or equal to 0.95 and a specificity of 1 [79]. The PCRS was thus found to be an efficient screening tool for assessing catatonia in adolescent and pediatric populations [79].

There has been a difference in time to treatment seen in acute catatonia and chronic catatonia. An observational study was performed on 34 patients with catatonic symptoms admitted to one hospital’s emergency department over a 2-year span [80]. Each patient was diagnosed by two psychiatrists as having symptoms of catatonia, examined for other psychiatric symptoms, and then treated accordingly. [80]. Researchers also communicated with members of each patient’s family to determine the length of time between the onset of symptoms of catatonia and the time at which each patient was admitted to the emergency department [80]. Patients were assigned to a grouping based on their underlying symptoms; the four groups included individuals with schizophrenic disorders, neuroleptic-induced disorders, mood disorders, and other pre-existing conditions [80]. A significant difference in the lapse in time between the onset of catatonic symptoms and the date of admittance to the emergency department was observed between each of the four groups (*p* < 0.05) [80]. It was observed that patients who experienced acute catatonic syndrome endured symptoms for less than 1.83 weeks on average before seeking treatment, while those who experienced chronic catatonic syndrome endured the associated symptoms for over 3.33 weeks on average before entering the emergency department [80]. These results indicate that a distinction of having experienced symptoms for 2–3 weeks before seeking treatment should be implicated to differentiate between patients experiencing chronic or acute catatonic symptoms [80].

A prospective one-year study analyzed the symptoms and outcomes of patients admitted to a psychiatric ward who were diagnosed with catatonia [81]. Throughout one year, catatonia was diagnosed on 15 occasions across 12 patients [81]. On each occasion, 1–2 mg of lorazepam was administered to the catatonic patient, after which the patient’s symptoms completely resolved in less than 2 h in 12 cases and partially resolved in 1 case [81]. This study’s findings suggest that for patients with catatonic episodes, the early identification and administration of lorazepam may be beneficial to the patient’s prognosis and may shorten the length of the patient’s hospitalization [81].

A trial was conducted to observe whether dopaminergic metabolites, Parkinsonian motions, and anxiety could be used to predict how a patient with acute catatonia would respond to short-term lorazepam administration [82]. Patients were treated with 2–4 mg of lorazepam and were monitored for 24 h [82]. Measurements were taken immediately before lorazepam administration and 24 h after administration [82]. Metabolic measurements included serum homovanillic acid (HVA) and serum 3-methoxy-4-hydroxyphenylglycol (MHPG); Parkinsonian motions were measured according to the Subjective Experiences of Psychosis Scale (SEPS) and the Abnormal Involuntary Movement Scale (AIMS), and anxiety was measured using the Hamilton Anxiety Scale (HAM-A) [82]. Before receiving treatment, patients who responded to short-term lorazepam treatment showed significantly increased serum HVA (*p* = 0.004), AIMS scores (*p* = 0.022), and HAM-A scores (*p* = 0.025) and significantly decreased SEPS scores (*p* = 0.049) relative to patients who were non-responsive [82]. These findings suggest that serum HVA, SEPS, AIMS, and HAM-A may be useful in determining whether a patient with acute catatonia is more likely to respond to short-term lorazepam treatment [82].

A cross-sectional study examined various associations as well as distinguished criteria between schizophrenia and catatonia [79]. The study included 225 patients meeting DSM-4 criteria for schizophrenia and catatonic symptoms [79]. A total of 72 of the patients met relatively more specific criteria adapted from the BFCRS for catatonia [79]. Three of the most common relevant symptoms of those who met the criteria for catatonia included mutism, grimacing, and portraying mannerisms [79]. It was observed that patients with more severe catatonia were more likely to exhibit symptoms of catatonia earlier in life and have relatively increased negative symptoms [79]. This study demonstrated that several scales, including the BFCRS, SANS, BPRS, Clinical Global Impression (CGI), Global Assessment Scale (GAS), and Nurses’ Observation Scale for Inpatient Evaluation (NOSIE (+)) can be used to distinguish non-catatonic from catatonic patients with chronic schizophrenia [79]. These findings indicate that despite similarities in symptoms, catatonia can be identified in patients independently from schizophrenia and is associated with a worse prognosis relative to non-catatonic patients with chronic schizophrenia [79].

### 5.3. Complications and Risk Factors

A case–control study was performed to identify risk factors associated with the development of neuroleptic malignant syndrome (NMS) in patients who had catatonia [80]. A total of 12individuals who met criteria for NMS and 24 individuals without NMS who were also treated with neuroleptics were included and compared in this study [80]. Previously hypothesized risk factors for NMS, such as elevated neuroleptic dose, were assessed along with novel possible risk factors such as catatonia and disorganization [81]. Each patient was examined by psychiatrists who monitored for the presence of identified potential risk factors for developing NMS [80]. Several statistically significant risk factors for NMS were verified in this study; these risk factors included catatonia, disorganization, and psychomotor agitation [80]. Behavioral risk factors identified in this study, including catatonia, should be closely monitored clinically in patients receiving neuroleptics to avoid the development of NMS [80].

### 5.4. Treatment

An open, cross-sectional study was performed in 16 African individuals ranging from 10 to 24 years of age to observe the therapeutic efficacy of lorazepam in treating catatonic patients with nodding syndrome (NS), which was described as a neurological syndrome characterized by episodes of repetitively dropping the head forward [80]. A variation of the BFCRS was used to evaluate symptoms of catatonia in each patient with NS [80]. Catatonic symptoms with the greatest average scores in these patients included mutism, grimacing, and stupor [80]. Patients were administered either 0.5 mg lorazepam or 1 mg lorazepam, depending on whether their body weight was less than or greater than 30 kg, respectively [80]. A reduction in a modified BFCRS score of greater than 50% was observed in six patients [80]. A dose of 0.5 to 1 mg lorazepam was found to reduce symptoms of catatonia in patients with NS in 13 of the 16 study participants [80]. Lorazepam was found to be an effective treatment for catatonic symptoms in patients with NS in this study. However, these findings may need to be replicated in larger-scale controlled experiments to verify their external validity [80].

A double-blind crossover study was performed to compare how oxazepam and lorazepam affect mutism and psychomotor retardation in psychiatric patients [82]. Patients were assessed for psychiatric symptoms and received a score of at least a 3 on the Bech–Rafaelsen Melancholia Rating Scale (BRMRS) criteria of the BPRS pertaining to psychomotor retardation on the first day of the study [82]. Patients were then randomly treated on the second day with either 2 mg lorazepam or 60 mg oxazepam and were treated with the opposite medication the following day [82]. Patients were assessed throughout each day for symptoms of psychomotor retardation using the BRMRS criteria of the BPRS as well as the 100 mm Visual Analog Scale (VAS) [82]. After the first day of treatment, 57% of the patients who received lorazepam first and 60% of the patients who received oxazepam first had an average improvement of at least 50% in their psychomotor retardation symptoms [82]. This study’s findings show that 3-hydroxybenzodiazepines, including both lorazepam and oxazepam, can effectively treat symptoms of catatonia. These findings also suggest that the effects of both lorazepam and oxazepam in the treatment of catatonia are facilitated by 3-hydroxybenzodiazepines binding to benzodiazepine receptors [82]. Table 1 summarizes the studies discussed in this section.

## 6. Conclusions

Catatonia is a complex condition with varying presentations and that is associated with multiple disorders, which can make recognition, diagnosis, and treatment a challenging process for healthcare professionals. Catatonic symptoms are often associated with various psychological and neurological disorders, including schizophrenia, neuroleptic malignant syndrome, and nodding syndrome. Patients with any of the symptoms of catatonia should be further evaluated for a catatonic syndrome secondary to their medical condition or independently of a psychiatric diagnosis. Although the etiology of catatonia is still unknown, diminished GABA signaling and abnormal OFC stimulation have been implicated in its pathophysiology.

The onset of catatonic symptoms and the presence of certain risk factors, such as elevated serum HVA, may help to predict whether a patient will respond to lorazepam. Benzodiazepines, specifically lorazepam, and ECT have been observed to be effective in treating acute catatonic symptoms associated with various mental disorders in both pediatric and adult populations. Early treatment of catatonia can reduce the risk of patients developing complications.

## Figures and Tables

**Table 1:** Clinical studies on diagnosis and treatment of Catatonia.

Author (Year)	Summary	Results	Conclusions
Benarous, Xavier et al. (2016)	A total of 138 psychiatric patients between the ages of 4 and 18 were used to evaluate measures of efficacy of the PCRS, including sensitivity, specificity, and ROC. A total of 88 patients met BFCRS criteria for catatonia, and 50 controls participated.	The PCRS exhibited a specificity of 1 and a sensitivity ≥0.95 in diagnosing patients with catatonia. The AUC for the ROC curve for patients treated with and without antipsychotics was ≥0.978.	PCRS was found to be a valid measure of catatonia in an adolescent/pediatric population.
Berardi, D. et al. (1998)	A total of 12 patients with a history of NMS and 24 controls were treated with neuroleptics and were monitored each day for the presence of extrapyramidal symptoms and pathological psychological symptoms.	Psychological symptoms such as disorganization (*p* < 0.002), confusion (*p* < 0.0006), and catatonia (*p* < 0.01) were identified as potential risk factors for NMS. Aspects of neuroleptic dose were also identified as potential risk factors.	Catatonia could increase one’s likelihood of developing NMS.
Conca, A. et al. (2003)	A total of 59 patients with catatonic symptoms were administered zuclopenthixol-acetate and were monitored using laboratory values including serum ferritin, iron, transferrin concentrations, body temperature, and other measures.	A total of 43 of the 59 patients developed a BTHR that lasted for ≥3 days on average. A total of 39.5% of the patients who developed a BTHR also developed ferropenia. A total of 32.2% of all catatonic patients treated with zuclopenthisol-acetate experienced ferropenia but did not develop signs of NMS.	Ferropenia may be associated with catatonia as well as an increased likelihood of experiencing a BTHR.
Huang, T. L. et al. (1999)	Families of 34 patients with catatonic symptoms that were admitted to Chang Gung Memorial Hospital at Linkou between January 1995 and March 1997 were consulted regarding the onset of the patient’s symptoms. Each patient was monitored and treated for catatonic symptoms.	The average timespan between the onset of catatonic symptoms and emergency department visit varied significantly among different types of psychological disorders (with catatonic symptoms). The majority of patients with acute catatonic syndrome had an onset of catatonic symptoms of <1.83 weeks before being admitted to the ER. Most patients with chronic catatonic syndrome had an onset of catatonic symptoms of >3.33 weeks before being admitted to the ER.	Acute or insidious onset of catatonic symptoms should be considered when treating patients with catatonia. The difference between acute or insidious onset should be considered as exhibiting catatonic symptoms for greater than or less than 2–3 weeks before receiving treatment.
Kakooza-Mwesige, Angelina et al. (2015)	A variation of the BFCRS was used to diagnose 16 patients between 10 and 24 years of age with NS. These 16 patients also met BFCRS criteria for catatonia. Effects of 0.5 or 1 mg of lorazepam (dosage determined by body weight of greater or less than 30 kg) on symptoms of NS and catatonia were monitored.	A total of 6 of the BFCRS scores in catatonic patients with NS were reduced by >50% after receiving treatment with lorazepam. After a single dose of 0.5 mg of lorazepam, scores were reduced in 10 of the 16 patients.	Lorazepam was shown to successfully treat symptoms of NS in patients between the ages of 10 and 24. This study’s small-scale findings indicate that lorazepam may serve as an effective treatment for symptoms of catatonia in patients with NS.
Northoff, G. et al. (1995)	A total of 18 patients diagnosed with acute catatonia received treatment with 2–4 mg of lorazepam and were monitored for dyskinetic motions, serum HVA concentration, and anxiety 24 h after receiving treatment.	Before treatment, short-term responsive patients had an average serum HVA concentration of 130.4 +/− 51.2 pmol/mL while non-responsive patients had an average serum HVA concentration of 73.2 +/− 40.5 pmol/mL. A total of 24 h after beginning treatment, short-term responsive patients received lower SEPS scores (*p* = 0.049) but higher AIMS scores (*p* = 0.022) and HAM-A scores (*p* = 0.025) relative to nonresponsive patients.	Serum HVA levels, presence of dyskinetic motions, and anxiety can be used to differentiate between catatonic patients that do or do not respond to short-term lorazepam treatment.
Richter, Andre et al. (2010)	A total of 6 patients diagnosed with catatonia and 16 healthy controls were administered either 1 mg lorazepam or placebo. fMRI scans were then performed on each participant while they were exposed to appropriate stimuli to trigger targeted brain activity. Each catatonic patient was also examined using the treatment that was not initially administered.	Catatonic patients who received lorazepam rather than placebo were found to have greater signal decreases within the ROIs of the OFC while triggered to experience negative emotions compared to healthy controls (*p* < 0.001).	Decreased GABAergic activity in the brain may have a role in the etiology of catatonia. Abnormal OFC function is observed in patients with catatonia.
Rosebush, P. I. et al. (1990)	A total of 12 patients were observed for catatonic symptoms over the span of one year. These catatonic patients were treated with 1–2 mg lorazepam when each patient experienced symptoms of catatonic syndrome.	A total of 15 cases of catatonic syndrome were observed in these 12 patients. The three most common signs of catatonia observed in these patients were mutism, staring, and immobility. A total of 66.67% of the patients in this study who benefited from lorazepam (1–2 mg) treatment suffered from CNS symptoms and not only from psychogenic catatonia. A total of 80% of the cases of catatonic syndrome were resolved in 2 h with lorazepam administration.	Early diagnosis and administration of lorazepam in catatonic patients may enhance prognosis and decrease length of hospitalization.
Schmider, J. et al. (1999)	A total of 21 patients with mutism and psychomotor retardation participated in this 3-day study. Patients were assigned a baseline assessment using the BPRS on day 1. Patients were then given 60 mg of oxazepam or 2 mg of lorazepam, alternating treatments between the 2nd and 3rd days. Each patient’s response to each treatment was assessed.	Both benzodiazepines were found to be beneficial in treating psychomotor retardation (*p* < 0.0001). The group that received oxazepam followed by lorazepam (10 patients) experienced greater relief of symptoms relative to the group of 7 patients who received lorazepam followed by oxazepam (*p* < 0.01). Each benzodiazepine relieved symptoms of catatonia in 19/21 patients. The two patients who did not respond to either benzodiazepine were later diagnosed with Parkinson’s Disease.	This study’s findings support the findings of other studies that lorazepam and oxazepam may both relieve symptoms of catatonia via agonistically binding to benzodiazepine receptors.
Ungvari, Gabor S. (2010)	A total of 15 patients diagnosed with schizophrenia according to DSM-4 criteria with catatonic symptoms participated in this 15-week trial. Each patient was daily given a placebo or 200 mg amineptine for 6 weeks and then received the alternate treatment for 6 weeks, separated by a 3-week intermission with no treatment, and was monitored for catatonic symptoms.	Amineptine was not found to have a significant effect on the symptoms of catatonia in each patient. No significant differences were observed between each treatment group (placebo received first or amineptine received first), and neither group showed a significant reduction in catatonic symptoms according to each clinical scale used (*p* > 0.35).	Dopaminergic pathways may not contribute significantly to symptoms of catatonia in schizophrenic patients.
Ungvari, Gabor S. et al. (1999)	A total of 18 patients with schizophrenia with chronic catatonic symptoms participated in this double-blind crossover trial. Patients received either placebo or lorazepam (6 mg daily) for 6 weeks then alternated treatments for 6 weeks after a wash-out period lasting 4 weeks. Symptoms of catatonia were monitored for each patient at weeks 0, 3, and 6 for each treatment using several clinical rating scales.	A significant difference was not observed between lorazepam or placebo treatment in the reduction of symptoms of catatonia across all clinical ratings. No patient experienced any clinically significant relief of catatonic symptoms after receiving either treatment based on BFCRS criteria.	The source of catatonia in schizophrenic patients with chronic catatonic symptoms may be caused by different pathophysiology than in schizophrenic patients with acute catatonic symptoms. Lorazepam was not found to be an effective treatment in schizophrenic patients with chronic catatonic symptoms.
Ungvari, Gabor S. et al. (2005)	A total of 274 patients between the ages of 18 and 55 years who had been diagnosed with chronic schizophrenia for over 5 years were assessed using numerous clinical rating scales for the severity of their psychiatric symptoms including symptoms of catatonia. These data were compiled and analyzed.	An increased BFCRS summed score for a schizophrenia patient with catatonia was associated with a higher SANS summed score (*p* < 0.001) and an earlier inception of catatonic symptoms (*p* = 0.002). The catatonic group had significantly different scores from the non-catatonic group of patients on assessments, including the BPRS (*p* = 0.0004), SANS (*p* < 0.001), CGI (*p* < 0.001), GAS (*p* = 0.001), and NOSIE (+) (*p* < 0.001).	Symptoms of catatonia can be distinguished in patients with chronic schizophrenia. The presence of catatonic symptoms in chronic schizophrenia patients is correlated with a worse prognosis than that of patients without catatonic symptoms.

## Data Availability

Data supporting the results above can be found on PubMed.
